# Experimental Investigation on the Diffusion Law of Polymer Slurry Grouted in Sand

**DOI:** 10.3390/polym14173635

**Published:** 2022-09-02

**Authors:** Zhenhua Li, Zihao Zhu, Yahong Zhao, Cong Zeng, Peng Zhang

**Affiliations:** 1 Faculty of Engineering, China University of Geosciences (Wuhan), Wuhan 430074, China; 2 Guangdong GDH Water Co., Ltd., Shenzhen 518000, China

**Keywords:** polymer slurry, chemical grouted sands, pneumatic grouting, diffusion laws

## Abstract

Polymer slurry is widely used in underground engineering treatment, but due to the concealed nature of underground projects, the diffusion pattern of slurry in the sand has been little studied. In this study, the basic physical properties of water-based polyurethane, oil-based polyurethane, and acrylate and epoxy resin were compared, and the performance of chemical grouted sands with different polymer slurry/sand mass ratios (PS/S) was tested. The higher the PS/S, the better the mechanical and impermeability properties of the chemical grouted sands. In this paper, water-based polyurethane was selected to carry out orthogonal tests on the diffusivity of slurry in sands. This experiment investigated the degree of influence of sand quality, grouting pressure and sand compactness on the diffusion of slurry in sands. The test results show that, in terms of factors affecting the final pressure of grouting, the sand density has the greatest influence, followed by the sand quality, and the grouting pressure is the smallest. In terms of slurry diffusibility, grouting pressure has the greatest influence, followed by sand compactness, and the sand quality is the smallest. The diffusion mechanism of slurry in the sand was deduced from the morphology of chemical grouted sands. Water-based polyurethane showed splitting-compression-penetration diffusion in sands of different grades, and the diffusion pattern of the slurry was not the same in low-pressure (1–1.5 MPa) grouting and high-pressure (2 MPa) grouting, and lateral splitting occurred in the case of high-pressure grouting diffusion.

## 1. Introduction

Underground urban buildings are an important infrastructure and component of cities, and their applications cover municipalities, water resources, transportation and electricity, which are the lifeblood of maintaining the normal operation of modern cities [1]. Due to the influence of objective factors such as the underground environment and material life, as well as the limitations of the design level and construction technology at the time, the underground projects built in the early days had local or even large-scale water seepage problems. Polymer slurry, considered to be a grouting material with excellent comprehensive properties [2], is now widely used in underground engineering to solve the problem of concrete structure seepage and water leakage and soil dehollowing. As underground engineering is a hidden project, most engineers only focus on the effect of governance, and there is little research on chemical grouted sands formed by the slurry at the back of the concrete structure.

Many scholars have conducted a lot of research on polymer slurries and the properties of chemical grouted sands formed by the slurry and the sand. Mohanad et al. summarized the current state of research on the use of expandable polyurethane resins for settlement compensation and lifting and strengthening the foundations of existing building sand structures, and summarized numerous polyurethane resin mechanical property testing studies [2]. Wang et al. have conducted many mechanical properties tests on polyurethane materials [3,4,5,6,7,8,9]. Liao analyzed the applicability of acrylate in seepage water treatment through cycle tests [10]. Anagnostopoulos has conducted many chemical grouted sands performance tests to study the effect of different epoxy resin/water ratios on the physical and mechanical properties of epoxy resin grouted sands [11,12,13,14,15,16]. Liu et al. found through experimental tests that the polyurethane grouted sands still had good shear properties after immersion, but the shear properties decreased with increasing immersion time [17,18,19,20]. Mohsen et al. have experimentally concluded that increasing relative dry density increased the elastic modulus of polyamide grouted sands in the same polyamide percent grouted sample [21]. Jin et al. used urea-formaldehyde resins and oxalate curing agents to produce chemical grouted sands of different particle sizes, the results of which showed that the coarser the particle size, the lower the uniaxial compressive strength [22]. Zang et al. investigated the effect of different segments in polyurethane on the mechanical properties of chemical grouted sands [23]. Norbaya et al. showed that the compressive strength of chemical grouted sands increases with the percentage of polyurethane content [24,25]. Wang et al. designed a urea resin grouting test in sands and demonstrated that the shape of the grouting sand is determined by splitting and compaction, while the size of the grouting sand shape is determined by infiltration. [26] Pan et al. showed by image processing analysis that the sand layer grouting has obvious common slurry diffusion patterns such as slurry enrichment, dominant path, cleavage action and subduction action [27]. Guo et al. studied the diffusion behavior of expanded polymer grouting materials in sand, gravel and sand-gravel mixtures through experimental and numerical analysis, showing that diffusion mainly depends on the pore and particle size of the medium [28]. Gao injected urea-formaldehyde resin and oxalic acid biofluid material into transparent soil to study the effect of using different grouting parameters on the diffusion of slurry and the formation of chemical grouted sands [29].

From the research of domestic and international experts and scholars in recent years, fruitful results have been achieved in terms of grouting materials and chemical grouted sands, but most of them focus on the influence of different factors on the mechanical properties of chemical grouted sands and the influence of different factors on the diffusion radius of the slurry, and there are very few studies on the seepage resistance of chemical grouted sands for underground engineering water leakage repair and the factors that significantly influence the diffusion pattern of the slurry. It is also difficult to describe the time-varying nature of the grouting process.

This paper mainly studies the grouting diffusion law of polymer slurry in sand, explores the significant factors affecting the slurry diffusion form, and deduces the time-varying process of slurry diffusion. In the second chapter of this paper, the performance tests of slurry solidified sand bodies are carried out to compare the basic physical properties of water-based polyurethane (WPU), oil-based polyurethane (OPU), acrylate and epoxy resin (EP), and to investigate the changes of mechanical properties and impermeability of chemical grouted sands with different polymer slurry/sand mass ratio (PS/S) of the four materials. In the third chapter of this paper, orthogonal tests on the influencing factors of slurry diffusion pattern are carried out to analyze the diffusion pattern of slurry in sand according to the diffusion pattern of chemical grouted sands, and to investigate the degree of influence of different sand qualities, different injection pressures and different soil compactness on the injectability and diffusion of slurry in the sand. Chapter 4 of this paper analyzes the morphology of chemical grouted sands according to Chapter 3, deduces the diffusion mechanism of the slurry at low to medium pressure (1–1.5 MPa) and high pressure (2 MPa), and determines the diffusion pattern of the slurry in the sand. Chapter 5 is the conclusion of this paper. The results of this experimental study and related discussions provide a theoretical basis and experimental knowledge for further exploration of the polymeric treatment of water seepage problems in underground engineering, which is of great practical significance for underground rehabilitation projects.

## 2. Basic Physical Properties Testing and Material Selection of Polymers

### 2.1. Material Testing

In the Chinese specification GB/T-50123 [30], the main materials used for leak plugging in underground projects are water-based polyurethane (WPU); oil-based polyurethane (OPU); acrylate and epoxy resin (EP); thus, these four materials were selected for testing and the basic physical properties of the slurry were tested according to the test method GB/T-50320 [31].

**Figure 1:**
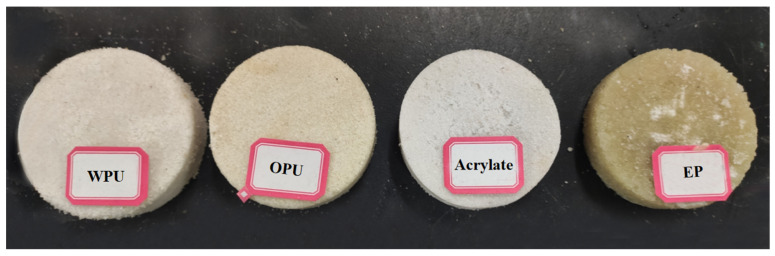
Chemical grouted sands impermeability test specimens.

Drawing on ASTM D4320M-09 [32] and ASTM D4219-08 [33], polymer slurry was mixed with 20 mesh size machined quartz sand and poured into the mold according to a certain slurry/sand mass ratio (PS/S) to form chemical grouted sands. Specimens with dimensions of 40 mm × 40 mm × 40 mm and φ61.8 mm × 40 mm were made for the unconfined compressive strength test and the permeability coefficient test, respectively, and the impermeability specimens are shown in Figure 1.

**Figure 2:**
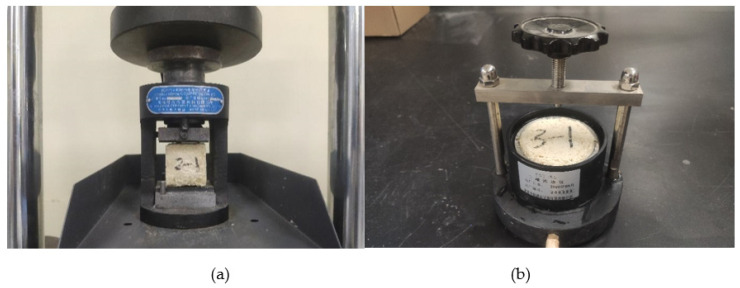
Chemical grouted sands performance test: (**a**) compressive strength test; (**b**) permeability coefficient test.

The performance test was carried out after the sample was placed for 72 h. The compressive strength test was carried out according to the GB/T-7671 [34]. The sample was put into the MTS universal testing machine and tested at a rate of 500 N/s. The permeability coefficient test was carried out according to the test method of GB/T-50123 [30]. The samples were placed in a TST-55 permeameter to test under variable head conditions. The performance test of chemical grouted sands is shown in Figure 2.

### 2.2. Test Results

**Figure 3:**
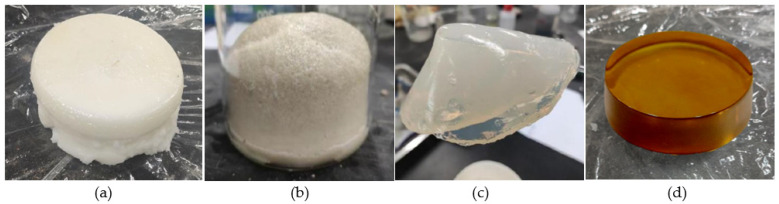
Polymer slurry gel or solidification state: (**a**) WPU; (**b**) OPU; (**c**) acrylate; (**d**) EP.

The basic physical properties of the polymer slurry are shown in Table 1 and the gel/solidification state of each slurry is shown in Figure 3. Both acrylates and epoxy resins are two-component polymers that require A and B components to be mixed and reacted in a certain ratio.

**Figure 4:**
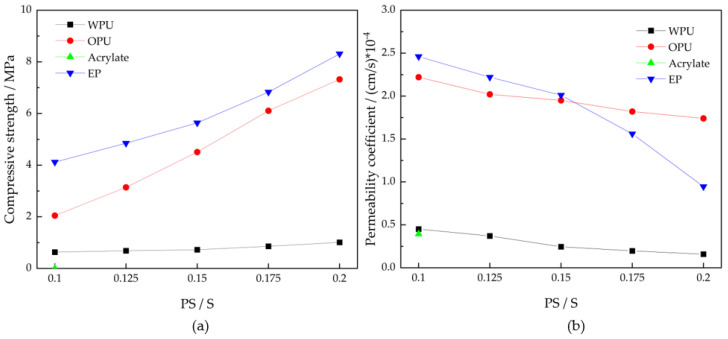
Chemical grouted sands performance change curve: (**a**) variation of compressive strength; (**b**) variation of permeability coefficient.

**Figure 5:**
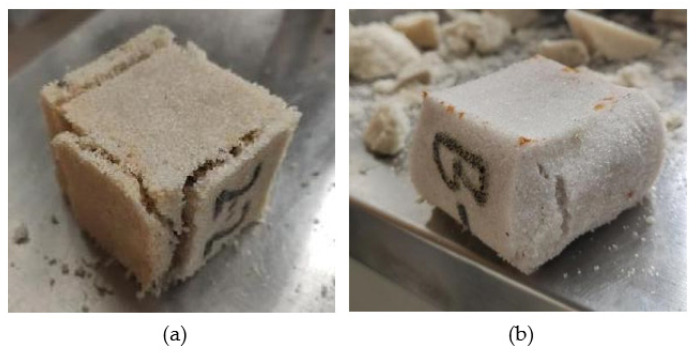
Chemical grouted sands destruction form: (**a**) EP grouted sands; (**b**) acrylate grouted sands.

The performance of chemical grouted sands varies with the PS/S mass ratio as shown in Figure 4. It can be seen that among the four materials, the higher the PS/S of WPU, OPU and EP, the higher the compressive strength of chemical grouted sands and the better the impermeability of chemical grouted sands. Acrylics cannot make chemical grouted sands with different PS/S due to fixed mixing reaction ratios. EP grouted sands have the highest strength, followed by the OPU grouted sands. EP grouted sands are brittle in the compressive test and the damage pattern is shown in Figure 5a, while the acrylate grouted sands are elastic in the compressive test and do not have strength; the damage pattern is shown in Figure 5b. WPU and acrylate grouted sands have the best impermeability performance, with values close to the impermeability coefficient of clay.

Among these four materials, WPU is a single-component polymer slurry, which can react directly with water and has the fastest reaction speed. WPU grouted sands have high impermeability and can better reflect the time when the slurry diffuses in the sand. Therefore, it is preferable to carry out sand diffusion tests with WPU.

## 3. Sand Diffusion Test

### 3.1. Experimental Design Options

The study shows that the main components of the loose aquifer are coarse sand, medium sand and fine sand. The main factors affecting the diffusion of sand are grouting pressure and the degree of loose sand. To explore which factors have the greatest influence on the diffusion of sand, it is necessary to design a comparative experiment. The orthogonal experiment can find the best experimental conditions and the characteristics of the greatest influence factor through the combination of fewer experiments. Therefore, a three-level, three-factor orthogonal trial was designed to carry out the study, and the experimental design is shown in Table 2.

### 3.2. Test Equipment and Materials

**Figure 6:**
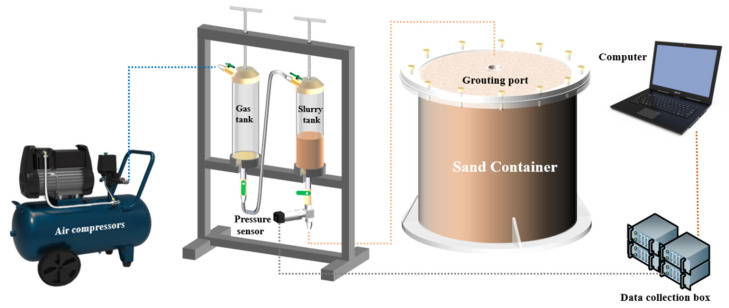
Model diagram of the test rig.

**Figure 7:**
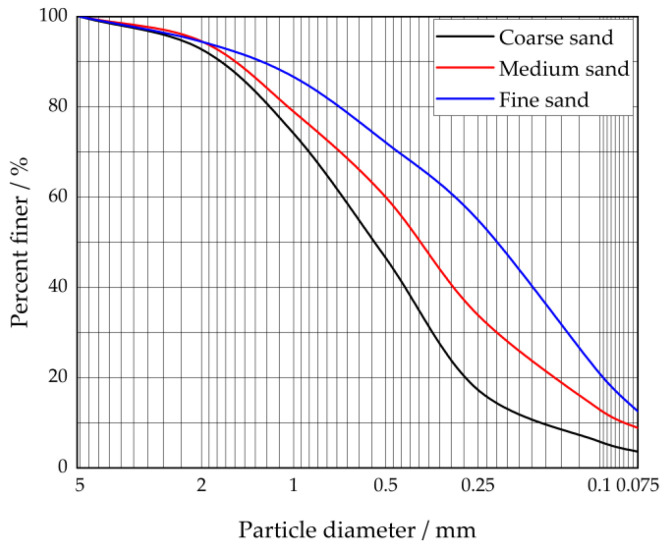
Test sand particle size gradation cumulative curve.

This test grouting method was carried out by pneumatic grouting. First, one must pour the slurry into the sand with a constant volume and pressure of the gas. Then, one must install a high-frequency pressure sensor to record the grouting data at the outlet of the slurry tank. The equipment and apparatus for this test are shown in Table 3, and the model diagram of the test set-up is shown in Figure 6. The geotechnical parameters of the sand used in this test were carried out according to the test method in GB/T-50123 [30]. The geotechnical parameters are shown in Table 4. The particle size gradation cumulative curve is shown in Figure 7.

### 3.3. Test Procedure

**Figure 8:**
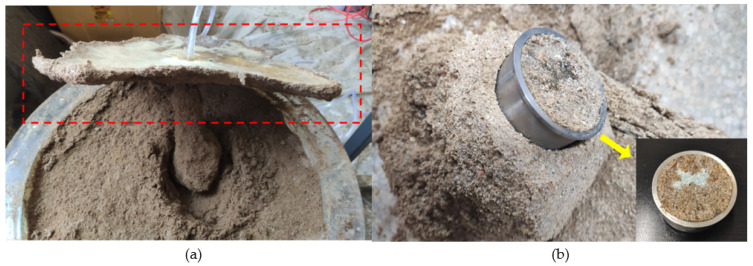
Obtaining a chemical grouted sands specimen: (**a**) excavation of chemical grouted sands; (**b**) sampling by cutting ring.

The test procedure steps are as follows. Fill the sand container with the test sand and use a rammer to control the compactness every 10 cm. After filling the sand, a 10 cm deep grouting pipe is buried. Cover the sealing ring and cover plate, and add 1.5 L of WPU to the slurry tank. Seal and connect the gas tank and slurry tank, then check the gas tightness of the device. The air compressor is used to pressurize the gas tank to the set pressure. Turn off the gas injection switch on the upper part of the gas tank, and open the lower switch of the slurry tank to start grouting. After grouting, clean the slurry tank. After waiting for 6 h, the chemical grouted sands formed by the coagulation of the slurry and sand are excavated. After cutting out the slurry-returning part of the chemical grouted sands in Figure 8a, measure their size, mass and volume. The chemical grouted sands of suitable size are sampled with a ring knife to test their permeability, as shown in Figure 8b.

## 4. Test Results and Analysis

### 4.1. Analysis of Grouting Pressure Data

**Figure 9:**
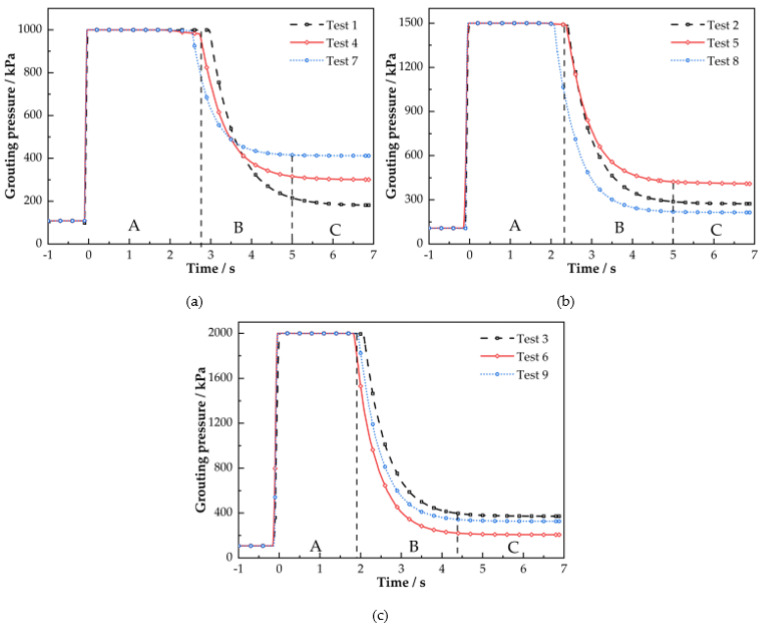
Grouting pressure curve: (**a**) test 1, 4, 7; (**b**) test 2, 5, 8; (**c**) test 3, 6, 9.

Variation in grouting pressure measured in this test is shown in Figure 9. As can be seen from Figure 9, the grouting pressure is mainly in three stages. The first stage A is the constant pressure grouting stage; the slurry enters the grouting pipe under the push of high-pressure gas. During this period, the pressure curve changes almost smoothly; the larger the starting pressure of grouting, the shorter the test grouting time. The second stage B is the diffusion stage; the slurry completely leaves the storage tank. At this point, the pressure curve begins to drop sharply as the pressure from the storage tank flows out. The third stage C is the stop grouting stage; the pressure of the grouting device is not enough to support the slurry to continue the diffusion. The pressure of the grouting pipe is consistent with the slurry pressure at the outlet of the grouting pipe. This value is the final pressure of grouting, which represents the injectable infiltration pressure of the sand and is related to the injectable starting pressure of the sand. At this point, the value changes slowly, except for one atmospheric pressure; the value is between 0.1 and 0.3 MPa. The grouting final pressure data were analyzed according to a single index orthogonal test and the results are listed in Table 5.

From the factor ranking in Table 5 and the *F*-value of the variance, it can be concluded that factor C (degree of sand compactness) has the most significant effect on the final pressure of grouting, followed by factor A (sand quality), and factor B (grouting pressure) has the least effect and the degree of effect is generally significant. The tighter the sand, the smaller the pore space inside the sand; the slurry is not easy to spread and the slurry pressure is not easy to release. Therefore, the initial grouting pressure of the slurry is mainly related to the degree of sand compaction.

### 4.2. Analysis of Slurry Diffusion Patterns

**Figure 10:**
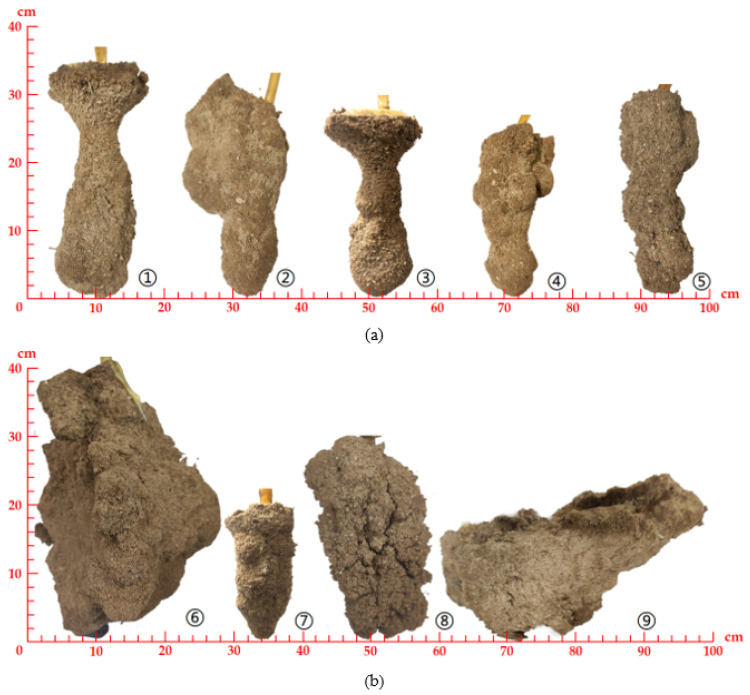
Chemical grouted sands chart: (**a**) test 1–5; (**b**) test 6–9.

The morphology of the chemical grouted sands in each group of the test is shown in Figure 10, and the various measurements of the test are shown in Table 6. The morphology of chemical grouted sands in tests 1–8 generally exhibits a long columnar shape. Some of the chemical grouted sands such as test 6 and 7 appear unevenly coral-like on the surface. The test 9 chemical grouted sand was condensed into a hemisphere because of the back slurry.

Except for test 6, all other test groups showed the phenomenon of returning slurry, while tests 3, 4, 5 and 7 were too small to take complete ring knife samples. The values of the impermeability coefficients of the chemical grouted sands that had been sampled were close. The mass and volume size are related to the diffusibility of polymer slurry effectively injected into the sand. The mass was analyzed according to a single indicator orthogonal test, the results of which are presented in Table 7.

From the factor ranking and the *F*-value of variance in the table, it can be concluded that the influence of grouting pressure is the most significant, followed by the degree of compactness, and the sand quality has the least influence. The higher the injection pressure, the higher the pressure on the sand interior, and the easier the slurry diffuses. The looser the sand, the lower the porosity and the more channels through which the slurry can diffuse. There is a small difference in the influence of sand quality on the test factors. It can be concluded that the relationship between diffusivity and sand depends mainly on the presence of diffusible channels in the sand.

### 4.3. Analysis of the Slurry Diffusion Mechanism

#### 4.3.1. Polymer Slurry Diffusion under Low and Medium Pressure Grouting Conditions

**Figure 11:**
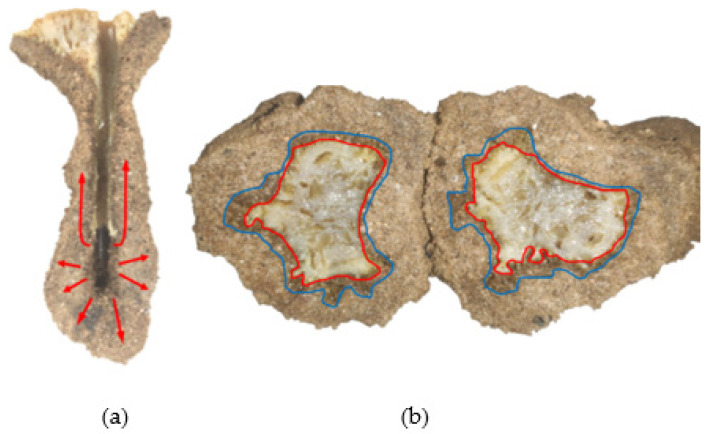
Section and profile of chemical grouted sands: (**a**) test 1 profile; (**b**) test 2 section.

Chemical grouted sands formed by grouting under the conditions of 1.0 and 1.5 MPa were cut with a cutter. We observed the diffusion pattern of the slurry in the sand in the lateral and vertical directions. The section and profile of chemical grouted sands are shown in Figure 11.

**Figure 12:**
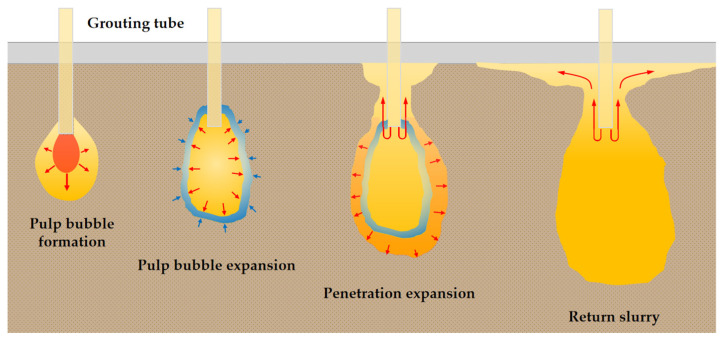
Schematic diagram of the slurry low-pressure diffusion process.

The area marked in red is the slurry bubble area; the slurry in the sand occurred in the longitudinal splitting diffusion phenomenon. The blue marked area is chemical grouted sands with more slurry components, and polymer slurry occurs in the sand as a compression diffusion phenomenon. The analysis of reasons for this is mainly: (1) the polymer slurry bubble occupies part of the space of the sand, resulting in the surrounding sand being compressed. (2) The kinetic energy of polymer slurry gradually dissipates in the diffusion process, and the diffusion speed slows down. Polymer slurry particles are deposited in the pores of the sand in the diffusion path, causing new resistance to the subsequently injected slurry. The slurry particles start to densely squeeze the surrounding sand. (3) WPU has its foaming properties and reacts with the sand moisture in the diffusion process to squeeze the sand. The remaining unmarked area is a homogeneous mixture of slurry and sand, which indicates that the slurry undergoes permeation diffusion in the sand. The sand skeleton hardly changes during the permeation diffusion. The slurry is continuously infiltrated and filled along the sand pore channels, and the slurry continuously reacts with water to gradually gel and solidify, cementing the loose sand particles into a whole. It can be inferred that the diffusion process of the slurry at low pressure (1–1.5 MPa) in the sand is divided into four main stages, as shown in Figure 12.

1. Polymer slurry bubble formation stage. The slurry forms pear-shaped slurry bubbles at the nozzle, and the slurry spreads rapidly along the sand voids. At the same time, part of the slurry pressure will displace the sand and cause longitudinal splitting.
2. Polymer slurry bubble expansion stage. As the slurry continues to spread, the slurry pressure gradually decreases, which is not enough to completely displace the sand. At this time, the slurry forms a compaction effect on the sand by compressing the sand, as shown in the blue marked area in Figure 12.
3. Penetration expansion stage. After compacting the sand, the slurry pressure continues to decrease, and the slurry penetrates and diffuses along the pores of the sand particles. At the same time, due to the decompression effect of diffusion, the slurry will look for a new diffusion channel. Due to the wall effect of the grouting pipe, the slurry will start to return to the slurry along the wall of the grouting pipe.
4. Returning polymer slurry stage. As the original slurry particles occupy the original diffusion path, the subsequently injected slurry needs to lose more slurry pressure to diffuse through the original diffusion path. When the slurry pressure cannot support the slurry diffusion in the original diffusion path, the slurry stops diffusing. The slurry starts to return along the wall of the grouting pipe and spreads to a more efficient path.

#### 4.3.2. Polymer Slurry Diffusion under High-Pressure Grouting Conditions

**Figure 13:**
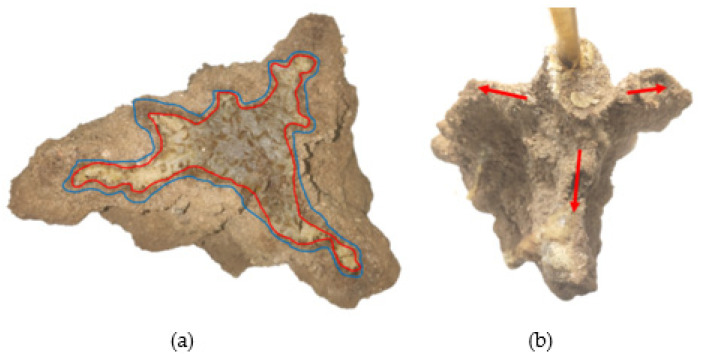
Test 6 chemical grouted sands: (**a**) sectional view; (**b**) top view.

The test 6 chemical grouted sand infused at 2 MPa high pressure was cut with a cutter and its section is shown in Figure 13a. Significant transverse splitting occurred in test 6 and its splitting path is shown in Figure 13b.

**Figure 14:**
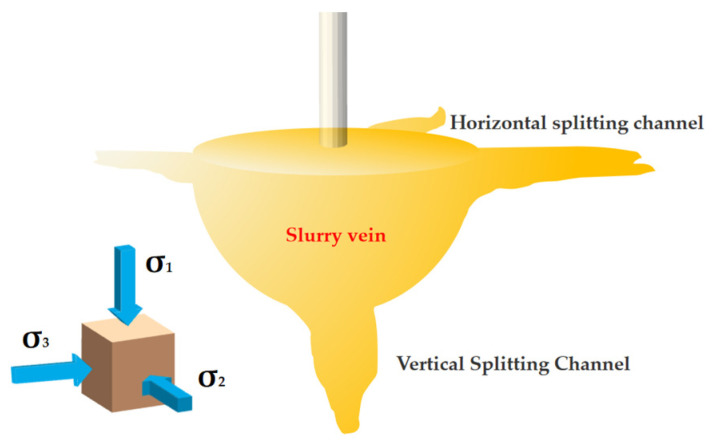
Schematic diagram of sand grouting splitting surface.

Observing Figure 13, combined with the stress analysis, the sand is subjected to ***σ*_1_**, ***σ*_2_** and ***σ*_3_** stresses in three directions. With the increasing slurry pressure, the stress state of the sand near the grouting hole gradually changes from a compressive stress state to a tensile stress state. Due to the low tensile strength of the sand layer, when the grouting pressure increases to the starting splitting pressure, the sand near the grouting hole will undergo tensile damage to form a splitting channel, and the slurry will enter the channel and expand continuously in the form of slurry veins. When the vertical stress ***σ*_1_** is greater than the horizontal stress ***σ*_2_** and ***σ*_3_**, vertical splitting will occur, as shown in Figure 14. As the slurry continues to fill, the slurry pressure inside the slurry bubble increases, and the direction and magnitude of the horizontal stress ***σ*_2_** and ***σ*_3_** will change. When it is greater than the vertical stress ***σ*_1_**, horizontal splitting will occur.

**Figure 15:**
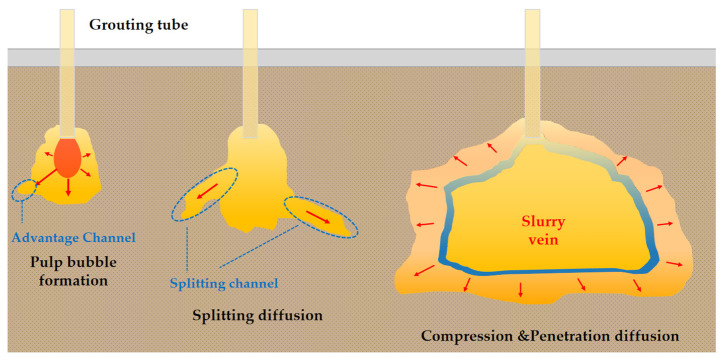
Schematic diagram of the slurry high-pressure diffusion process.

It is inferred that there are three main stages of lateral splitting and spreading of the slurry at high pressure, as shown in Figure 15.

1. Polymer slurry bubble formation stage. Polymer slurry forms pear-shaped slurry bubbles at the mouth of the pipe and longitudinal splitting occurs while the slurry under pressure seeks uniform and porous diffusion paths in the sand to form advantage channels.
2. Splitting diffusion stage. With the continuous replenishment of the slurry, the internal energy of the slurry bubble increases. The slurry will follow the dominant channel to form a larger splitting channel and continue to displace the sand to form slurry veins.
3. Compression and penetration diffusion stage. As polymer slurry diffusion energy gradually diminishes, the polymer slurry diffuses first through the compacted sand, followed by permeable diffusion along the pores of the sand, until the energy is so diminished that it cannot advance.

**Figure 16:**
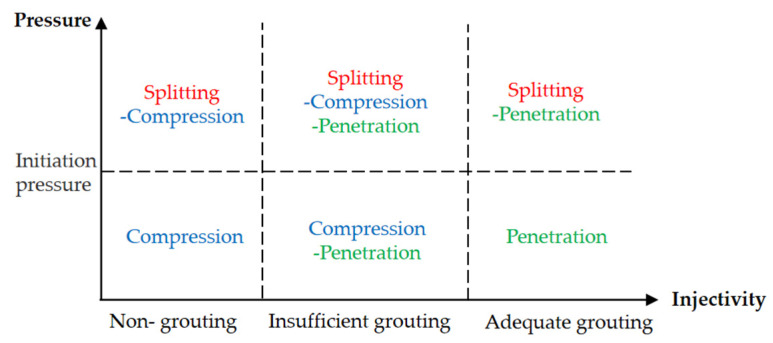
Division of slurry diffusion mode.

According to the diffusion theory [35], the six patterns of slurry diffusion are divided as shown in Figure 16. Judging from the test results, regardless of the conditions, the injectability of this test belongs to insufficient grouting and the diffusion pattern belongs to the splitting-compression-penetration pattern.

## 5. Conclusions

In this study, the basic physical properties of water-based polyurethane (WPU), oil-based polyurethane (OPU), and acrylate and epoxy resin (EP) were compared, and the compressive strength and permeability coefficient of chemical grouted sands were tested for different polymer slurry/sand mass ratios (PS/S). In addition, the degree of influence of different sand qualities, different grouting pressures and different sand compactness on the diffusion of slurry in sands was investigated by three-factor, three-level orthogonal tests. The diffusion mechanism of slurry in the sand was deduced according to the morphology of chemical grouted sands. The results obtained in this paper are as follows.

1. The higher the PS/S of polymer chemical grouted sands, the better its compressive performance and seepage resistance. OPU grouted sands and EP grouted sands have similar performance; EP grouted sands have the highest compressive strength, the compressive strength of PS/S 0.2 is more than 8 MPa, WPU grouted sands have the best seepage resistance, the permeability coefficient of PS/S 0.2 is less than 2.5 × 10^−5^ cm/s.
2. For the sand injectability factors, the degree of sand compactness has the greatest influence; the looser the sand, the greater the number of sand pore channels, the smaller the sand injectable permeability pressure. The test grouting final pressure value was 0.1–0.3 MPa. For the sand diffusion morphology factors, the grouting pressure has the greatest influence; the higher the grouting pressure, the more the sand is injected by the slurry, and the formation of chemical grouted sands mass and volume is also larger. Sand quality has less influence on the injectability of the slurry and the diffusion pattern of the sand.
3. WPUs in different grading of sand grouting mode all belong to splitting-compression-penetration diffusion; belong to incomplete grouting; the slurry diffusion form mostly shows long column type; the slurry diffusion process in low- and medium-pressure (1–1.5 MPa) grouting is mainly divided into polymer slurry bubble formation stage, polymer slurry bubble expansion stage, permeation expansion stage and returning polymer slurry stage. The slurry diffusion in high-pressure (2 MPa) grouting appears as lateral splitting; the diffusion process is mainly divided into polymer slurry bubble formation stage; splitting diffusion stage; compression and penetration diffusion stage.

## Figures and Tables

**Table 1:** Basic physical properties of polymer slurry.

Materials	Density/(g/cm^3^)	Viscosity/mPa·s	Gel/Setting Time/s	Foaming Rate
**WPU**	1.04	360	22	560
**OPU**	1.17	584	530	1789
**A** **CRYLATE**	Component A: 0.95	Component A: 23	38	-
	Component B: 0.91	Component B: 22		
**EP**	Component A: 1.06	Component A: 335	1800	-
	Component B: 1.03	Component B: 560		

**Table 2:** Orthogonal test table.

Test Number	Sand Quality (A)	Grouting Pressure (B)	Sand Compactness (C)
**1**	(1) Coarse sand	(1) 1.0 MPa	(1) Loose
**2**	(1) Coarse sand	(2) 1.5 MPa	(2) Moderate
**3**	(1) Coarse sand	(3) 2.0 MPa	(3) Tight
**4**	(2) Medium sand	(1) 1.0 MPa	(2) Moderate
**5**	(2) Medium sand	(2) 1.5 MPa	(3) Tight
**6**	(2) Medium sand	(3) 2.0 MPa	(1) Loose
**7**	(3) Fine sand	(1) 1.0 MPa	(3) Tight
**8**	(3) Fine sand	(2) 1.5 MPa	(1) Loose
**9**	(3) Fine sand	(3) 2.0 MPa	(2) Moderate

**Table 3:** Test equipment and instruments.

Instruments	Properties
Acrylic round sand container	Internal diameter 570 mm, height 600 mm
Gas tank	Volume 4 L
Slurry tank	Volume 4 L
NI acquisition systems	50 data acquisitions in 1 s
High-frequency pressure sensor	Measurement range 0–4 MPa
Rammer	Impact pressure 0.5 MPa
Air compressors	Measurement range 0–2 MPa

**Table 4:** Geotechnical parameters of sand.

	Natural Density/(g/cm^3^)	Moisture Content (%)	Porosity Ratio	Permeability Coefficient/(cm/s)
Coarse sand	1.61	17.5	0.97	2.05 × 10^−3^
Medium sand	1.72	20.3	0.89	4.80 × 10^−4^
Fine sand	1.74	22.4	0.81	3.20 × 10^−4^

**Table 5:** Analysis table for the orthogonal test of final pressure of slurry injection.

	Level	Sand Quality (A)	Grouting Pressure (B)	Sand Compactness (C)
**Final Pressure of Slurry Injection**		*m_1j_*	*m_2j_*	*m_3j_*
	1	275.19	297.86	200.67
	2	305.79	299.13	300.24
	3	317.56	301.54	397.63
	*D_ij_*	42.37	3.67	196.96
**Factor Ranking**	C > A > B			
**Analysis of Variance *F*-Value**		16.41	0.12	332.69
**Level of Impact**		Significant	General	Very significant

**Table 6:** Chemical grouted sands measurement data.

Test Number	Quality/g	Volume/cm^3^	Permeability Coefficient/cm/s
**1**	1491.8	740	5.02 × 10^−5^
**2**	2816.0	1430	5.28 × 10^−5^
**3**	1891.4	920	-
**4**	1154.2	540	-
**5**	1313.1	670	-
**6**	4942.8	2300	4.50 × 10^−5^
**7**	800.2	380	-
**8**	2748.1	1420	4.76 × 10^−5^
**9**	3592.1	1830	4.84 × 10^−5^

**Table 7:** Analysis table for the orthogonal test of chemical grouted sands mass.

	Level	Sand Quality (A)	Grouting Pressure (B)	Sand Compactness (C)
**Chemical Grouted Sands Quality**		*m_1j_*	*m_2j_*	*m_3j_*
	1	2066.40	1148.73	3060.90
	2	2470.03	2292.40	2520.77
	3	2380.13	3475.43	1334.90
	*D_ij_*	403.63	2326.70	1726
**Factor Ranking**	B > C > A			
**Analysis of Variance *F*-Value**		0.19	5.76	3.32

## Data Availability

Not applicable.
